# *Plasmodium falciparum pfhrp2* and *pfhrp3* Gene Deletions from Persons with Symptomatic Malaria Infection in Ethiopia, Kenya, Madagascar, and Rwanda

**DOI:** 10.3201/eid2803.211499

**Published:** 2022-03

**Authors:** Eric Rogier, Jessica N. McCaffery, Doug Nace, Samaly Souza Svigel, Ashenafi Assefa, Jimee Hwang, Simon Kariuki, Aaron M. Samuels, Nelli Westercamp, Arsène Ratsimbasoa, Milijaona Randrianarivelojosia, Aline Uwimana, Venkatachalam Udhayakumar, Eric S. Halsey

**Affiliations:** Centers for Disease Control and Prevention, Atlanta, Georgia, USA (E. Rogier, J.N. McCaffery, D. Nace, S.S. Svigel, A.M. Samuels, N. Westercamp, V. Udhayakumar);; Ethiopia Public Health Institute, Addis Ababa, Ethiopia (A. Assefa);; Centers for Disease Control and Prevention, Atlanta, Georgia, USA (J. Hwang, E.S. Halsey);; Centre for Global Health Research, Kenya Medical Research Institute, Kisumu, Kenya (S. Kariuki);; Centers for Disease Control and Prevention, Kisumu (A.M. Samuels);; Madagascar National Malaria Control Program, Antananarivo, Madagascar (A. Ratsimbasoa);; Institut Pasteur de Madagascar, Antananarivo (M. Randrianarivelojosia);; Université de Toliara, Toliara, Madagascar (M. Randrianarivelojosia);; Rwanda Biomedical Center, Kigali, Rwanda (A. Uwimana)

**Keywords:** malaria, sub-Saharan Africa, *Plasmodium falciparum*, *pfhrp2*, *pfhrp3*, gene deletion, rapid diagnostic tests, vector-borne infections, therapeutic efficacy studies, histidine-rich protein 2, Ethiopia, Kenya, Madagascar, Rwanda

## Abstract

Histidine-rich protein 2 (HRP2)–based rapid diagnostic tests detect *Plasmodium falciparum* malaria and are used throughout sub-Saharan Africa. However, deletions in the *pfhrp2* and related *pfhrp3* (*pfhrp2/3*) genes threaten use of these tests. Therapeutic efficacy studies (TESs) enroll persons with symptomatic *P. falciparum* infection. We screened TES samples collected during 2016–2018 in Ethiopia, Kenya, Rwanda, and Madagascar for HRP2/3, pan-*Plasmodium* lactate dehydrogenase, and pan-*Plasmodium* aldolase antigen levels and selected samples with low levels of HRP2/3 for *pfhrp2/3* genotyping. We observed deletion of *pfhrp3* in samples from all countries except Kenya. Single-gene deletions in *pfhrp2* were observed in 1.4% (95% CI 0.2%–4.8%) of Ethiopia samples and in 0.6% (95% CI 0.2%–1.6%) of Madagascar samples, and dual *pfhrp2/3* deletions were noted in 2.0% (95% CI 0.4%–5.9%) of Ethiopia samples. Although this study was not powered for precise prevalence estimates, evaluating TES samples revealed a low prevalence of *pfhrp2/3* deletions in most sites.

The World Health Organization (WHO) estimates there were 228 million cases of malaria in 2019, which resulted in 409,000 deaths; >90% of these deaths occurred in sub-Saharan Africa (*1*). Although all 4 human malaria *Plasmodium* species are present in Africa, *Plasmodium falciparum* accounts for most symptomatic infections (*1*). After the WHO recommended confirming *Plasmodium* infection before initiating treatment (*2*), malaria rapid diagnostic tests (RDTs) have been widely deployed because of their ease of use and high diagnostic sensitivity for symptomatic infection (*3*–*5*). The histidine-rich protein 2 (HRP2) antigen is produced exclusively by *P. falciparum* parasites, and RDTs detecting this antigen provide a practical tool for diagnosis in both healthcare and community settings (*1*,*3*,*6*) and have revolutionized the diagnosis of malaria throughout Africa.

HRP2-based RDTs are an accurate diagnostic tool because HRP2 is abundantly expressed during the erythrocytic stage of *P. falciparum* infection (*6*). The *pfhrp3* gene is paralogous to *pfhrp2* and has a high level of similarity in both gene sequence and the expressed histidine-rich protein 3 (HRP3) antigen, although the HRP3 antigen is substantially shorter in length (*7*,*8*). However, because of common epitopes on both antigens, they jointly contribute to an overall HRP2-based RDT positive result or laboratory assay signal (*6*,*9*). In many areas of the world, *P. falciparum* variants have been identified with loss-of-function mutations or complete deletions of the *pfhrp2* and *pfhrp3* (*pfhrp2/3*) genes, which lead to false-negative RDT results (*6*,*10*). Multiple countries in sub-Saharan Africa have reported the presence of *P. falciparum* with deletions in these genes (*9*,*11*–*16*), although only Eritrea and Djibouti have reported a prevalence of >5% among isolates from symptomatic infections (*17*,*18*).

WHO recommends routine therapeutic efficacy studies (TESs) approximately every 2 years in malaria-endemic countries to assess antimalarial drug efficacy, and US President’s Malaria Initiative funding ensures these studies routinely occur in many countries throughout sub-Saharan Africa (*19*). According to established WHO protocol (*20*), symptomatic patients with uncomplicated *P. falciparum* malaria are enrolled in healthcare facilities after infection is verified by light microscopy examination of a blood smear. In addition, on the day of enrollment and subsequent follow-up days, a blood sample from a finger prick is dried on filter paper to form a dried blood spot (DBS) to monitor chemotherapeutic efficacy and test for putative drug resistance genetic markers (*19*). TESs are often implemented at multiple sites in a country because efficacy might vary depending on local endemicity, *P. falciparum* haplotypes, and antimalarial use.

We investigated deletions in *pfhrp2/3* genes by using samples from TESs in Ethiopia (2017), Kenya (2016–2017), Madagascar (2018), and Rwanda (2018). DBS samples from day of enrollment were subjected to multiplex antigen detection and subsequent PCR assays if *pfhrp2/3* genotyping was warranted on the basis of the antigen profile.

## Materials and Methods

### Therapeutic Efficacy Studies

This study focuses on TESs in 4 countries: Ethiopia (enrollment during September–December 2017) (*21*), Kenya (enrollment during June 2016–March 2017) (*22*), Madagascar (enrollment during May–September 2018) (*23*), and Rwanda (enrollment during May–December 2018) (*24*). Specific site information and enrollment criteria are provided for each TES by the indicated reference. Of note, enrollment criteria in the Madagascar TES included a positive HRP2-based RDT result. CDC human subjects review for laboratory analyses for all TES samples were determined independently for each study: Ethiopia as engaged research (#6892.0), Rwanda as program evaluation (#2018-060), Madagascar as nonengaged research (#2018-435), and Kenya as engaged research (#6696.0).

### Bead-Based Multiplex Assay for Malaria Antigen Detection

All DBS samples were processed and analyzed within 1 year of creation. We performed elution of whole blood from DBS samples and the bead-based multiplex assay for malaria antigen detection as described previously (*25*) (Appendix). Differences among parasite densities or antigen levels were assessed by Student t test for unequal variances using the log-transformed data.

### Selection of Samples for Further Genetic Assays

Using the strategy reported previously (*26*,*27*), we selected samples for further genetic assays on the basis of the relationship between the 2 pan-*Plasmodium* antigens (aldolase and lactate dehydrogenase [LDH]) and the HRP2/3 signal. Samples were selected if they completely lacked an assay signal for HRP2/3 or if the assay signal for HRP2/3 was atypically lower compared with the level of pAldolase or pLDH antigens.

We extracted total genomic DNA from 6-mm punches of selected DBS samples by using the QIAGEN DNA extraction kit (QIAGEN, https://www.qiagen.com) following the manufacturer’s instructions for blood dried on filter paper. The DNA was eluted in 150 μL of elution buffer, aliquoted, and stored at −20°C until further use.

### Photo-Induced Electron Transfer PCR and Genotyping for *pfmsp1, pfmsp2, pfhrp2, and pfhrp3*

After DNA extraction, we performed photo-induced electron transfer PCR as described previously (*28*) to ensure presence of *P. falciparum* DNA. We used nested PCR to genotype *pfmsp1, pfmsp2,* and *pfhrp3* as described previously (*29*). For *pfhrp2* genotyping, we performed PCR on these samples under conditions described previously (*30*). Results for *pfhrp2/3* genotyping were only reported if both *pfmsp1* and *pfmsp2* (both single-copy genes in the *P. falciparum* genome) were successfully amplified for a DNA sample (*31*).

## Results

**Figure 1:**
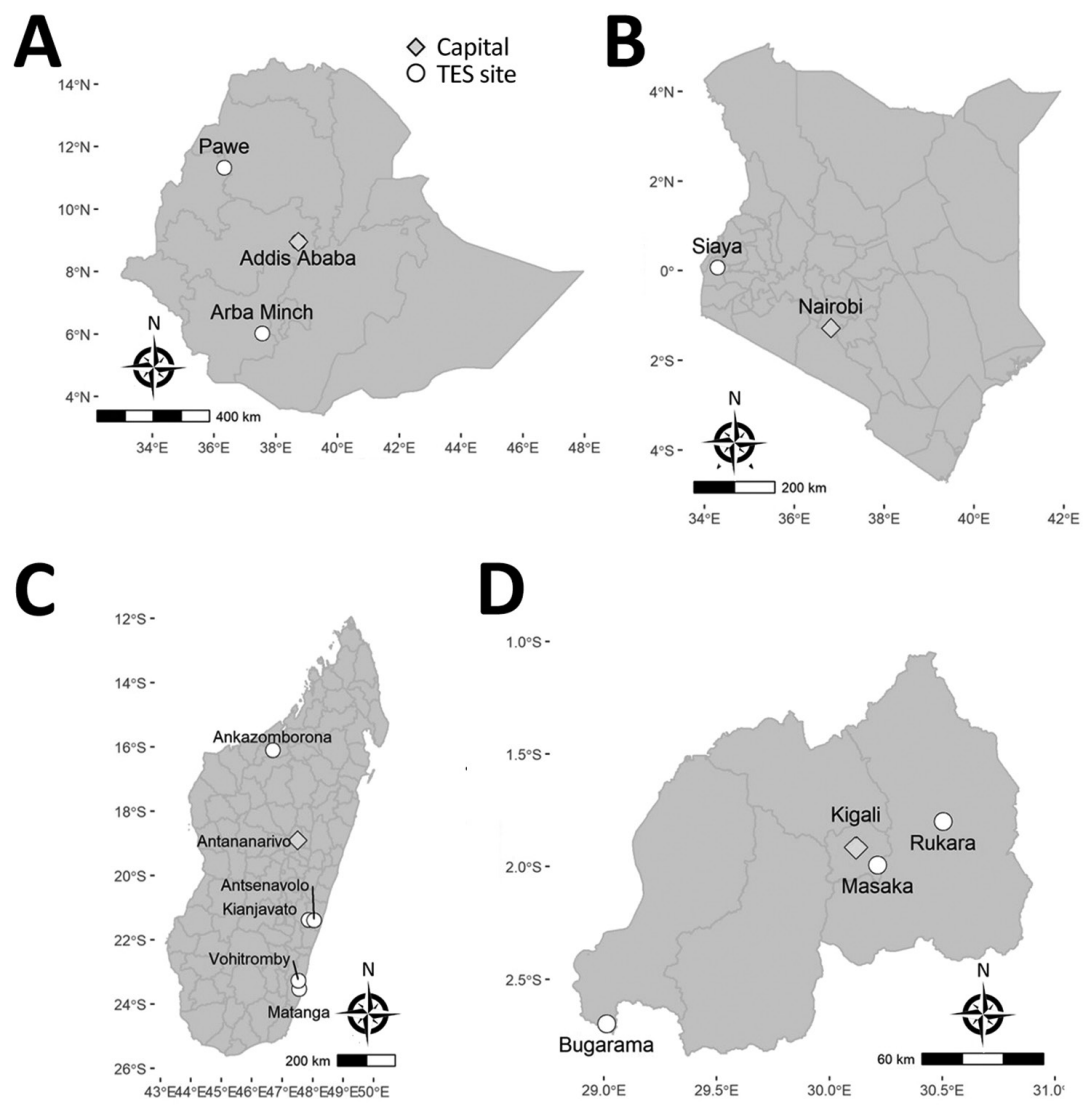
Location of TES sites where *Plasmodium falciparum* malaria-infected participants were enrolled, Ethopia, Kenya, Madagascar, and Rwanda, 2016–2018. A) Ethiopia, B) Kenya, C) Madagascar, D) Rwanda. Circles indciate study sites and diamonds the country capitals. Scale bars are unique to each map. TES, therapeutic efficacy study.

The Kenya 2016–2017 TES had the fewest number of sites at 1, followed by the Ethiopia 2017 TES at 2, the Rwanda 2018 TES at 3, and the Madagascar 2018 TES at 5 (Figure 1). The number of participants providing DBS samples from each site at enrollment varied for each of the 4 countries and ranged from a low of 15 participants at the Arba Minch, Ethiopia, site to a high of 332 participants at the Siaya, Kenya, site (Table 1). Reflecting the different enrollment criteria for each TES, the median and range of participant ages were unique to each TES; median age was 18.0 years in Ethiopia, 2.7 years in Kenya, 7.0 years in Madagascar, and 3.3 years in Rwanda. Enrollment by sex was mostly equal for the Kenya, Madagascar, and Rwanda TESs; 48.8% (Kenya), 47.1% (Madagascar), and 50.9% (Rwanda) of participants providing DBS samples in these studies were women. Enrollment of women in the Ethiopia TES was notably lower at 32.9%.

**Table 1:** Countries and study sites for each therapeutic efficacy study enrolling *Plasmodium falciparum* malaria–infected participants, 2016–2018

Country and study site	No. specimens at enrollment	Median age (range), y	Sex, % F
Ethiopia	147	18.0 (1–65)	32.9
Arba Minch	15	19.5 (10–54)	50.0
Pawe	132	18.0 (1–65)	31.3
Kenya			
Siaya	332	2.7 (0.5–4.9)	48.8
Madagascar	620	7.0 (0.2–15)	47.1
Ankazomborona	168	8.3 (1.5–15)	41.7
Antsenavolo	54	6.0 (0.2–14)	53.7
Kianjavato	116	9.0 (0.3–15)	46.6
Matanga	172	5.0 (0.3–15)	48.3
Vohitromby	110	7.0 (1–15)	50.9
Rwanda	218	3.3 (0.7–4.8)	50.9
Bugarama	88	3.3 (0.8–4.8)	52.3
Masaka	42	3.3 (0.8–4.0)	54.8
Rukara	88	3.1 (0.7–4.8)	46.6

The antigen screening methodology provided phenotypic rationale for categorizing the infecting *P. falciparum* as a high producer of HRP2/3 antigens or a HRP2/3 low-producer requiring subsequent characterization though genetic assays (*25*,*26*). Correlation of antigen assay signal with parasite density (as determined by microscopy during enrollment for each TES) (Appendix Figure 1) showed that the 2 pan-*Plasmodium* antigens displayed a moderate correlation with microscopy-estimated *P. falciparum* parasite density, whereas the HRP2 antigen showed higher variability, as seen previously (*25*). We compared the pAldolase and pLDH assay signal to the HRP2 assay signal for all samples from each of the 4 countries and chose select samples for DNA extraction (Table 2; Appendix Figures 2–5). Ethiopia had the highest percentage (n = 21, 14.3% of all DBS samples) of samples selected for DNA extraction and PCR genotyping, followed by Rwanda (n = 16, 7.3%), Madagascar (n = 25, 4.0%), and Kenya (n = 7, 2.1%).

**Table 2:** *Plasmodium falciparum* malaria–infected participant DBS samples with atypical HRP2 levels selected for further genomic assays, Ethiopia, Kenya, Madagascar, and Rwanda*

Country and study site	No. specimens at enrollment	No. specimens selected for genetic assays (%)	No. selected on pAldolase ratio only	No. selected on pLDH ratio only	No. selected on ratio to both
Ethiopia	147	21 (14.3)	4	7	10
Arba Minch	15	2 (13.3)	1	1	0
Pawe	132	19 (14.4)	3	6	10
Kenya					
Siaya	332	7 (2.1)	1	1	5
Madagascar	620	25 (4.0)	7	10	8
Ankazomborona	168	11 (6.5)	4	2	5
Antsenavolo	54	6 (11.1)	2	4	0
Kianjavato	116	1 (0.9)	0	1	0
Matanga	172	3 (1.7)	0	2	1
Vohitromby	110	4 (3.6)	1	1	2
Rwanda	218	16 (7.3)	5	6	5
Bugarama	88	9 (10.2)	2	4	3
Masaka	42	2 (4.8)	0	1	1
Rukara	88	5 (5.6)	3	1	1

*DBS, dried blood spot; HRP2, histidine-rich protein 2; pAldolase, pan-*Plasmodium* aldolase; pLDH, pan-*Plasmodium* lactate dehydrogenase.

**Figure 2:**
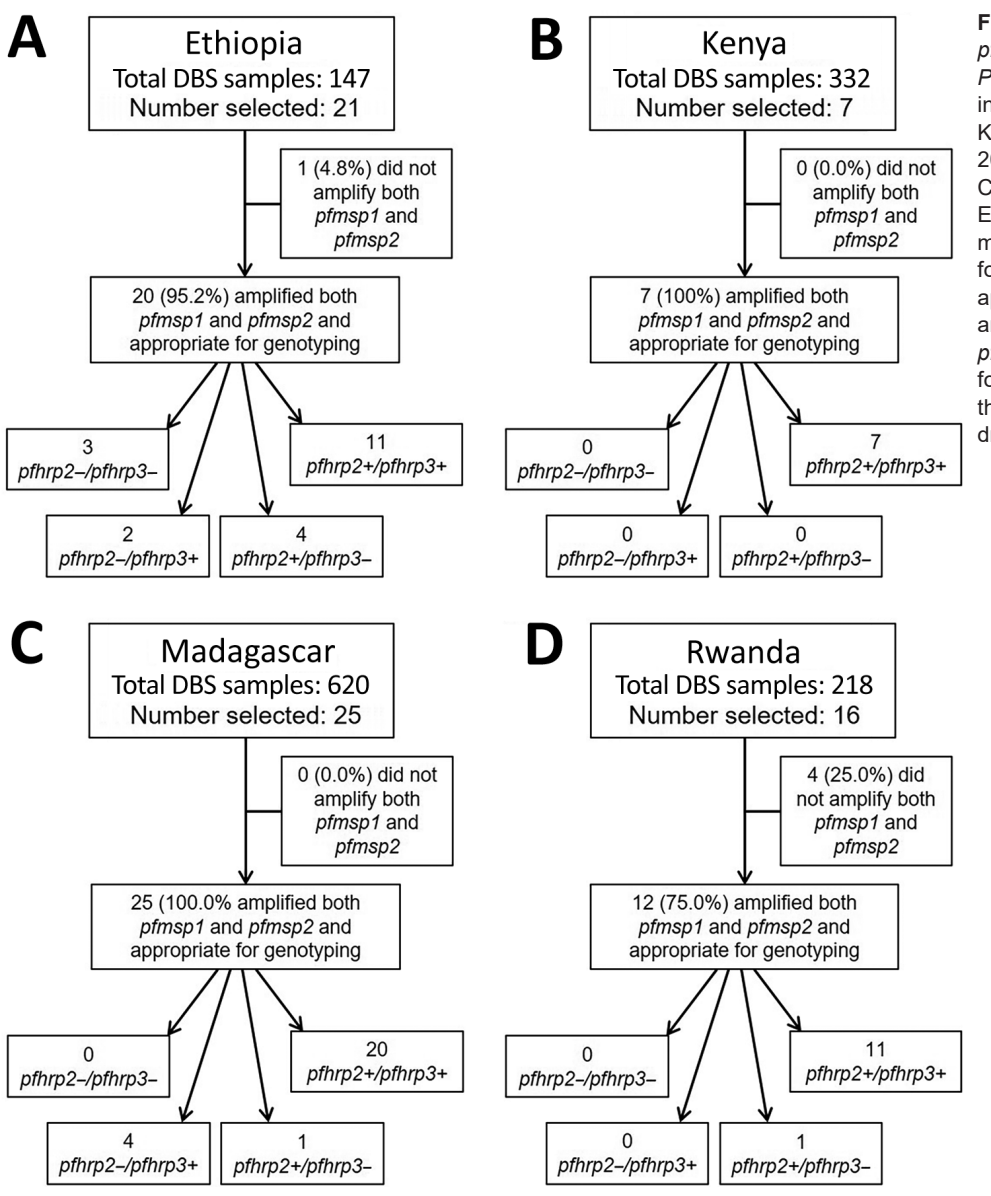
Results for *pfhrp2* and *pfhrp3* genotyping for DBSs from *Plasmodium falciparum* malaria-infected participants, Ethopia, Kenya, Madagascar, and Rwanda, 2016–2018. A) Ethiopia, B) Kenya, C) Madagascar, D) Rwanda. Each flowchart outlines how many specimens were selected for genotyping, how many were appropriate for genotyping (by amplification of both *pfmsp1* and *pfmsp2*), and genotyping results for presence (+) or absence (–) of the *pfhrp2* and *pfhrp3* genes. DBS, dried blood sample.

After initial sample selection for genotyping, we evaluated DNA quantity and quality appropriate for genotyping by amplification of both *pfmsp1* and *pfmsp2* genes. Final *pfhrp2/3* genotyping results were evaluated for amplification of (+) or failure to amplify (–) these 2 different gene targets (Figure 2). Only samples from which both the single-copy *pfmsp1* and *pfmsp2* genes were successfully amplified had *pfhrp2/3* genotype reported (*31*); from all selected samples, only 1 sample from Ethiopia and 4 samples from Rwanda were unsuccessfully amplified for these control genes. Most selected samples (76.6%) showed a wild-type genotype of *pfhrp2+/pfhrp3+*. Single gene deletions were observed in samples from 3 countries: single gene *pfhrp2* deletions from Ethiopia and Madagascar, and single gene *pfhrp3* deletions from Ethiopia, Madagascar, and Rwanda (Table 3). The *pfhrp2*–*/pfhrp3*– double deletion genotype was observed only in Ethiopia; 3 of the 20 samples selected, all from the Pawe site, showed this genotype. Because the *pfhrp3* nested PCR includes a reaction for an exon 1-to-2 spanning primer and a separate reaction for an exon 2 primer, a nested PCR reaction could have amplified one of these targets and not the other if the gene was not fully deleted from the genome. For 9 samples classified as negative for the *pfhrp3* gene, both of these nested PCR targets failed to amplify in all, with the exception of a single sample from Ethiopia (exon 1–2 target did not amplify and exon 2 target did; the sample was positive for the *pfhrp2* gene).

**Table 3:** Deletion genotypes by individual therapeutic efficacy study sites, Ethiopia, Kenya, Madagascar, and Rwanda

Country and study site	No. specimens at enrollment*	No. (%) specimens detected with *pfhrp2*–/*pfhrp3*–	No. (%) specimens detected with *pfhrp2*–/*pfhrp3*+	No. (%) specimens detected with *pfhrp2*+/*pfhrp3*–
Ethiopia				
Arba Minch	15	0	0	2 (13.3)
Pawe	132	3 (2.3)	2 (1.5)	2 (1.5)
Kenya				
Siaya	332	0	0	0
Madagascar				
Ankazomborona	168	0	3 (1.8)	1 (0.6)
Antsenavolo	54	0	0	0
Kianjavato	116	0	0	0
Matanga	172	0	0	0
Vohitromby	110	0	1 (0.9)	0
Rwanda				
Bugarama	88	0	0	1 (1.1)
Masaka	42	0	0	0
Rukara	88	0	0	0

*Percentages may underestimate the actual amount of deleted parasites because not all samples were genotyped, rather only those found to initially have depressed histidine-rich protein 2 levels. All samples with high histidine-rich protein 2 signal assumed to be from wild-type infections.

**Figure 3:**
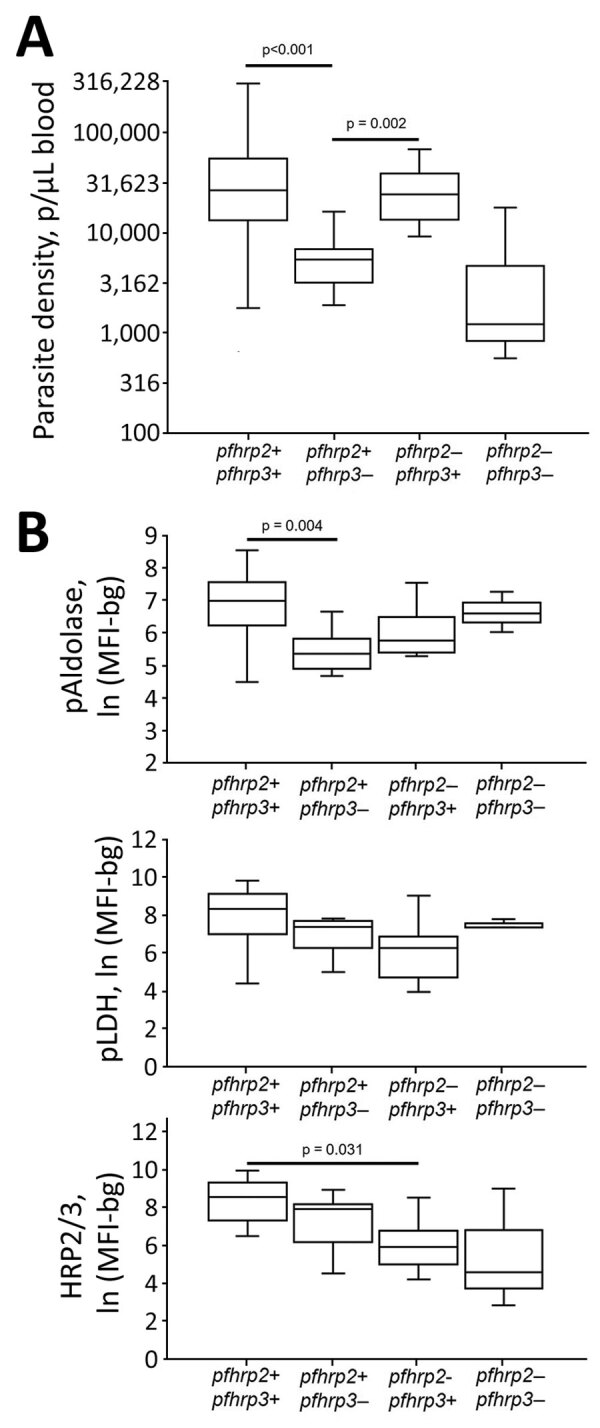
*Plasmodium falciparum* parasite density and antigen levels by *pfhrp2/3* genotype in study of *Plasmodium falciparum* malaria-infected participants, Ethopia, Kenya, Madagascar, and Rwanda, 2016–2018. A) Peripheral blood parasite density as determined by light microscopy. B) Log-transformed assay signal to pAldolase, pLDH, and HRP2/3 antigens. Boxes display interquartile range, horizontal lines within boxes indicate medians, and whiskers indicate 1.5× interquartile range. Significant differences in means are indicated with corresponding p values. Within each plot, all other differences among genotypes did not reach statistical significance at α = 0.05. HRP2/3, histidine-rich protein 2/3; ln (MFI-bg), log-transformed median fluorescence intensity minus background value; pAldolase, pan-*Plasmodium* aldolase; pLDH, pan-*Plasmodium* lactate dehydrogenase.

In an exploratory analysis of the 64 samples that were successfully genotyped, different *pfhrp2/3* genotype combinations showed significant differences in microscopy-estimated parasite densities (Figure 3, panel A). In comparison to wild-type parasites, significantly lower parasite densities were observed in infections with *P. falciparum* lacking the *pfhrp3* gene alone. Parasites lacking the *pfhrp2* gene alone showed significantly higher mean parasite densities when compared with the *pfhrp3* single-deleted infections. To link the phenotypic data of antigen expression with the *pfhrp2/3* genotypic data, we plotted the antigen detection assay signal by genotype for the 64 total samples that underwent successful genotyping. Assay signals for pan-*Plasmodium* aldolase, pan-*Plasmodium* LDH, and HRP2/3 by the 4 potential combinations of *pfhrp2/3* genotypes are given (Figure 3, panel B). With loss of either of the *pfhrp2* or *pfhrp3* genes and loss of both, no overall trend was observed for changes in pAldolase or pLDH signal, although the numbers of each of these genotypes were small. However, we observed a lower HRP2/3 assay signal with either the loss of the *pfhrp3* gene or the *pfhrp2* gene; the lowest mean HRP2/3 assay signal occurred when both genes were absent.

## Discussion

Deletion of the *pfhrp2* and *pfhrp3* genes poses a threat to the accuracy of HRP2-based RDT diagnosis of *P. falciparum* malaria, and parasites with deletions in one or both these genes have now been found in numerous countries (*6*,*10*). By far, most malaria cases in Africa are caused by *P. falciparum*, and the presence of these deletion genotypes in many countries throughout the continent poses an additional challenge to malaria control because of false-negative diagnostic results (*1*). Most countries in Africa have adopted the HRP2-based RDT as a pragmatic and sensitive diagnostic tool and the only *P. falciparum*-specific diagnostic test available in many settings. Loss of this tool would be a substantial setback to accurate monitoring of malaria case incidence within a country and to achieving the goal of universal confirmation of malaria infection before administrating antimalarials (*3*).

In this study, we sought to identify the presence of deletions in either the *pfhrp2* or *pfhrp3* genes from samples collected during routine TESs that enroll participants with microscopically confirmed *P. falciparum* infection. The primary objective of a TES is to assess in vivo efficacy of antimalarials, and some studies have also investigated the presence of *P. falciparum* drug resistance genetic markers (*19*). Because DBS samples are collected for many of these TESs, residual patient samples represent a convenience sampling of known *P. falciparum* infections with estimated parasite densities, and enrollment at healthcare facilities conforms to the WHO *pfhrp2* deletion guidance to sample symptomatic patients (*32*). Quantitative detection of malaria antigens in these DBS samples not only enables the confirmation of the presence or absence of HRP2, HRP3, or both in the patient’s blood sample, it also enables the simultaneous detection of other *Plasmodium* antigens for comparison. For these 4 TESs, a total of 1,317 DBS samples were available, and performing genetic characterization for all these samples would have required a large time and financial commitment. However, by initially employing a low-cost, high-throughput antigen screening step, fewer samples can be carefully selected for more definitive investigation into production of these RDT targets (*25*–*27*). This strategy of phenotypic screening and genetic confirmation is not unique for the TES sampling design and has also been used for healthcare facility (*25*,*27*) and community (*26*) surveys. Further exploration of this strategy with large datasets is needed throughout global *P. falciparum* populations to determine the overall accuracy of this methodology and its ability to generalize antigen levels with deletions of *pfhrp2* and *pfhrp3*.

Many TESs seek to enroll participants at multiple sites throughout a country to gain a more geographically representative sampling of *P. falciparum* for in vivo efficacy estimates. Of the data presented in this study, 3 of the 4 countries had multiple enrollment sites; only Kenya enrolled persons from just 1 site. Ultimately, high global variation has been observed in *pfhrp2* gene sequences (*7*,*33*,*34*), and deletions in the *pfhrp2* and *pfhrp3* genes can arise de novo in a *P. falciparum* population (*18*,*35*). Therefore, presence (or absence) of these gene deletions could not be accurately ascertained for an entire country by sampling a limited number of sites. Recent WHO guidance recommended enrolling from >10 health facilities per province to estimate whether *pfhrp2* deletions exceed 5% of all *P. falciparum* infections for a country (*32*). Because TESs do not enroll at many study sites, the data presented in this study do not provide country-representative or even precise local estimates of gene deletion prevalence, but they generate a data signal to point toward the presence of deletions at a site of a previous TES. Troublesome data signals generated from TES samples could be followed up with a more thorough study, such as the WHO-recommended approach of enrolling from a minimum of 10 health facilities per province to estimate whether *pfhrp2* deletions exceed 5% of all *P. falciparum* infections for a country. A benefit of this sampling design is that TESs are routinely performed in countries that receive support from the US President’s Malaria Initiativeevery 2–3 years (*19*); consistently collecting quantitative antigen data from these sample sets will provide a longitudinal approach to better identify emerging deletion genotypes in a country.

Samples with an absence of both the *pfhrp2* and *pfhrp3* are of greatest concern because these 2 genes express the only antigen targets recognized by an HRP2-based RDT. For the 69 samples selected for genotyping on the basis of antigen profile, only 3 double-deletions were noted, all arising from the Pawe study site in the Benishangul-Gumuz region in northeastern Ethiopia. As an external evaluation activity, persons enrolling in the Ethiopia TES were also tested by HRP2-based RDTs, and of the 3 persons with double-deleted *P. falciparum* infections, 2 tested negative by the HRP2 band on the RDT. These same 2 persons had a complete absence of HRP2/3 antigens by the bead-based assay, whereas the third double-deleted infection had an HRP2/3 antigen concentration in blood of 27.5 ng/mL. An additional 2 samples from Pawe were also found to be deleted for the *pfhrp2* gene alone (both of these persons were HRP2-RDT positive), meaning of the 132 total *P. falciparum* isolates available from Pawe, 3.8% (95% CI 1.2%–8.6%) showed a deletion of the *pfhrp2* gene. Multiple recent reports have uncovered the presence of *pfhrp2/3* deletions in central (*36*) and northern (*37*,*38*) Ethiopia at levels above the 5% WHO recommendation to reevaluate national RDT selection (*32*). The data presented in this study do not attempt to provide a prevalence estimate of single- or double-deletion *P. falciparum* genotypes in Ethiopia, but they add to the growing evidence of the pervasiveness of these parasites lacking *pfhrp2* in the country by demonstrating their presence in 2017.

Deletions in *pfhrp2* were also observed in samples from Madagascar, a country with numerous haplotypes circulating according to previous studies (*39*,*40*). Even with the identification of *pfhrp2* deletions in Madagascar, these cases represent a very small proportion (4 of 620, 0.6%) of all *P. falciparum*–infected children providing DBS samples for this study. Three of the 4 *pfhrp2*-deleted samples came from the Ankazomborona study site in the northern part of the country, which could provide rationale for further investigation of deletions in this part of Madagascar. Of note, the Madagascar TES had enrollment criteria of positivity to both microscopy and HRP2-based RDT. Because infections in persons with double-deleted parasites would likely have been excluded from enrollment, these data should be taken in that context. 

Separate investigations have (*9*) and have not (*7*) detected *pfhrp2* and *pfhrp3* deletions in Kenya. From the 332 DBS samples available from the 2016–2017 Kenya TES, most *P. falciparum* infections produced high amounts of HRP2/3 antigens, and no phenotypic or genotypic evidence was seen for gene deletions of these targets. A single report from Rwanda also identified nonamplification of the *pfhrp2* gene from microscopically positive *P. falciparum* infections, although the primers were only targeting the exon 2 of the gene (*13*), which is not crucial for antigen expression. In this study, no deletions of the *pfhrp2* gene were identified in Rwanda, and only 1 *P. falciparum* isolate was found with a *pfhrp3* deletion.

Existence of HRP2/3 antigens in a blood sample does not necessarily indicate that the currently infecting *P. falciparum* parasite possesses functioning *pfhrp2/3* genes. The bead assay limit of detection is ≈10 pg/mL, and HRP2 antigen can remain in blood for months after successful treatment of a *P. falciparum* infection (*6*,*41*). A person could therefore be actively infected with a deleted strain but have HRP2 antigen in their blood from a previous infection (although the levels would be expected to be atypically low in this scenario). Because of the phenotypic selection criteria outlined in this study, infections with high levels of HRP2/3 would not be selected for genotyping but might still harbor parasites with deletions of the *pfhrp2/3* genes and would not be captured, although this possibility is likely low. If deleted parasites were more likely to induce asymptomatic or less symptomatic infections, this enrollment criteria in healthcare facilities would lead to underestimating actual deletion prevalence in a population, although data have not demonstrated this effect. Simultaneous infection with multiple *P. falciparum* haplotypes was also not investigated in this study, so the presence of deleted parasites could be masked by the presence of wild-type parasites in the same host (*42*). These 2 scenarios would be more probable in a higher-transmission setting, where the likelihood for residual HRP2/3, as well as higher multiplicity of infection and more frequent infections, would be more common. In addition, the genetic assays used in this study attempted to simply amplify a region of DNA and do not provide information regarding potential loss-of-function point mutations or other genetic scenarios which would cause these 2 antigens not to be expressed. Because antigen degradation might occur in DBS samples over time, quantitative antigen detection should occur as soon as possible.

In conclusion, with appropriate patient consent, screening samples that were previously collected for routine TESs for *pfhrp2* and *pfhrp3* deletions represent a useful convenience sampling of persons with symptomatic and microscopically confirmed *P. falciparum* infection. These phenotypic and genotypic data provide information for a country to evaluate whether these genotypes exist and promote a basis for more targeted future surveys to obtain precise point estimates of prevalence.

Appendix Additional information about *Plasmodium falciparum pfhrp2* and *pfhrp3* gene deletions from persons with symptomatic malaria infection in Ethiopia, Kenya, Madagascar, and Rwanda.

## References

[1] World Health Organization. World malaria report 2020. Geneva: The Organization; 2020.

[2] World Health Organization. Guidelines for the treatment of malaria: 2nd edition. Geneva: The Organization; 2010.

[3] World Health Organization. Universal access to malaria diagnostic testing: an operational manual. Geneva: The Organization; 2011.

[4] Harvey SA, Bell D. How to use a rapid diagnostic test (Generic Pf): a guide for training at the village and clinic level. Geneva: World Health Organization; 2008.

[5] Plucinski M, Dimbu R, Candrinho B, Colborn J, Badiane A, Ndiaye D, et al. Malaria surveys using rapid diagnostic tests and validation of results using post hoc quantification of *Plasmodium falciparum* histidine-rich protein 2. Malar J. 2017;16:451. 10.1186/s12936-017-2101-8 29115966 PMC5678810

[6] Poti KE, Sullivan DJ, Dondorp AM, Woodrow CJ. HRP2: transforming malaria diagnosis, but with caveats. Trends Parasitol. 2020;36:112–26. 10.1016/j.pt.2019.12.004 31848119

[7] Nderu D, Kimani F, Thiong’o K, Karanja E, Akinyi M, Too E, et al. *Plasmodium falciparum* histidine-rich protein (PfHRP2 and 3) diversity in Western and Coastal Kenya. Sci Rep. 2019;9:1709. 10.1038/s41598-018-38175-1 30737461 PMC6368535

[8] Fontecha G, Pinto A, Escobar D, Matamoros G, Ortiz B. Genetic variability of *Plasmodium falciparum* histidine-rich proteins 2 and 3 in Central America. Malar J. 2019;18:31. 10.1186/s12936-019-2668-3 30704496 PMC6357481

[9] Beshir KB, Sepúlveda N, Bharmal J, Robinson A, Mwanguzi J, Busula AO, et al. *Plasmodium falciparum parasites* with histidine-rich protein 2 (*pfhrp2*) and *pfhrp3* gene deletions in two endemic regions of Kenya. Sci Rep. 2017;7:14718. 10.1038/s41598-017-15031-2 29116127 PMC5677122

[10] Thomson R, Parr JB, Cheng Q, Chenet S, Perkins M, Cunningham J. Prevalence of *Plasmodium falciparum* lacking histidine-rich proteins 2 and 3: a systematic review. Bull World Health Organ. 2020;98:558–568F. 10.2471/BLT.20.250621 32773901 PMC7411324

[11] Koita OA, Doumbo OK, Ouattara A, Tall LK, Konaré A, Diakité M, et al. False-negative rapid diagnostic tests for malaria and deletion of the histidine-rich repeat region of the hrp2 gene. Am J Trop Med Hyg. 2012;86:194–8. 10.4269/ajtmh.2012.10-0665 22302847 PMC3269266

[12] Wurtz N, Fall B, Bui K, Pascual A, Fall M, Camara C, et al. *Pfhrp2* and *pfhrp3* polymorphisms in *Plasmodium falciparum* isolates from Dakar, Senegal: impact on rapid malaria diagnostic tests. Malar J. 2013;12:34. 10.1186/1475-2875-12-34 23347727 PMC3571878

[13] Kozycki CT, Umulisa N, Rulisa S, Mwikarago EI, Musabyimana JP, Habimana JP, et al. False-negative malaria rapid diagnostic tests in Rwanda: impact of *Plasmodium falciparum* isolates lacking *hrp2* and declining malaria transmission. Malar J. 2017;16:123. 10.1186/s12936-017-1768-1 28320390 PMC5359811

[14] Funwei R, Nderu D, Nguetse CN, Thomas BN, Falade CO, Velavan TP, et al. Molecular surveillance of *pfhrp2* and *pfhrp3* genes deletion in *Plasmodium falciparum* isolates and the implications for rapid diagnostic tests in Nigeria. Acta Trop. 2019;196:121–5. 10.1016/j.actatropica.2019.05.016 31103699

[15] Parr JB, Verity R, Doctor SM, Janko M, Carey-Ewend K, Turman BJ, et al. *Pfhrp2*-deleted *Plasmodium falciparum* parasites in the Democratic Republic of the Congo: a national cross-sectional survey. J Infect Dis. 2017;216:36–44. 28177502 10.1093/infdis/jiw538 PMC6279174

[16] Thomson R, Beshir KB, Cunningham J, Baiden F, Bharmal J, Bruxvoort KJ, et al. *pfhrp2* and *pfhrp3* gene deletions that affect malaria rapid diagnostic tests for *Plasmodium falciparum*: analysis of archived blood samples from 3 African countries. J Infect Dis. 2019;220:1444–52. 10.1093/infdis/jiz335 31249999 PMC6761929

[17] Iriart X, Menard S, Chauvin P, Mohamed HS, Charpentier E, Mohamed MA, et al. Misdiagnosis of imported *falciparum* malaria from African areas due to an increased prevalence of *pfhrp2/pfhrp3* gene deletion: the Djibouti case. Emerg Microbes Infect. 2020;9:1984–7. 10.1080/22221751.2020.1815590 32869688 PMC7534257

[18] Berhane A, Anderson K, Mihreteab S, Gresty K, Rogier E, Mohamed S, et al. Major threat to malaria control programs by *Plasmodium falciparum* lacking histidine-rich protein 2, Eritrea. Emerg Infect Dis. 2018;24:462–70. 10.3201/eid2403.171723 29460730 PMC5823352

[19] Halsey ES, Venkatesan M, Plucinski MM, Talundzic E, Lucchi NW, Zhou Z, et al. Capacity Development through the US President’s Malaria Initiative-Supported Antimalarial Resistance Monitoring in Africa Network. Emerg Infect Dis. 2017;23:23. 10.3201/eid2313.170366 29155671 PMC5711327

[20] World Health Organization. Methods for surveillance of antimalarial drug efficacy. Geneva: The Organization; 2009.

[21] Leonard CM, Mohammed H, Tadesse M, McCaffery JN, Nace D, Halsey ES, et al. Missed *Plasmodium falciparum* and *Plasmodium vivax* mixed infections in Ethiopia threaten malaria elimination. Am J Top Med Hyg. 2021 Nov 30 [Epub ahead of print]. 10.4269/ajtmh.21-0796 PMC8832938 34847530

[22] Chebore W, Zhou Z, Westercamp N, Otieno K, Shi YP, Sergent SB, et al. Assessment of molecular markers of anti-malarial drug resistance among children participating in a therapeutic efficacy study in western Kenya. Malar J. 2020;19:291. 10.1186/s12936-020-03358-7 32795367 PMC7427724

[23] Dentinger CM, Rakotomanga TA, Rakotondrandriana A, Rakotoarisoa A, Rason MA, Moriarty LF, et al. Efficacy of artesunate-amodiaquine and artemether-lumefantrine for uncomplicated *Plasmodium falciparum* malaria in Madagascar, 2018. Malar J. 2021;20:432. 10.1186/s12936-021-03935-4 34732201 PMC8565026

[24] Uwimana A, Umulisa N, Venkatesan M, Svigel SS, Zhou Z, Munyaneza T, et al. Association of *Plasmodium falciparum kelch13* R561H genotypes with delayed parasite clearance in Rwanda: an open-label, single-arm, multicentre, therapeutic efficacy study. Lancet Infect Dis. 2021;21:1120–8. 10.1016/S1473-3099(21)00142-0 33864801 PMC10202849

[25] Plucinski MM, Herman C, Jones S, Dimbu R, Fortes F, Ljolje D, et al. Screening for *Pfhrp2/3*-deleted *Plasmodium falciparum*, non-*falciparum*, and low-density malaria infections by a multiplex antigen assay. J Infect Dis. 2019;219:437–47. 10.1093/infdis/jiy525 30202972 PMC6325347

[26] Bakari C, Jones S, Subramaniam G, Mandara CI, Chiduo MG, Rumisha S, et al. Community-based surveys for *Plasmodium falciparum pfhrp2* and *pfhrp3* gene deletions in selected regions of mainland Tanzania. Malar J. 2020;19:391. 10.1186/s12936-020-03459-3 33148255 PMC7640459

[27] Herman C, Huber CS, Jones S, Steinhardt L, Plucinski MM, Lemoine JF, et al. Multiplex malaria antigen detection by bead-based assay and molecular confirmation by PCR shows no evidence of *Pfhrp2* and *Pfhrp3* deletion in Haiti. Malar J. 2019;18:380. 10.1186/s12936-019-3010-9 31775743 PMC6882344

[28] Lucchi NW, Narayanan J, Karell MA, Xayavong M, Kariuki S, DaSilva AJ, et al. Molecular diagnosis of malaria by photo-induced electron transfer fluorogenic primers: PET-PCR. PLoS One. 2013;8:e56677. 10.1371/journal.pone.0056677 23437209 PMC3577666

[29] Abdallah JF, Okoth SA, Fontecha GA, Torres RE, Banegas EI, Matute ML, et al. Prevalence of *pfhrp2* and *pfhrp3* gene deletions in Puerto Lempira, Honduras. Malar J. 2015;14:19. 10.1186/s12936-014-0537-7 25604310 PMC4308922

[30] Jones S, Subramaniam G, Plucinski MM, Patel D, Padilla J, Aidoo M, et al. One-step PCR: A novel protocol for determination of *pfhrp2* deletion status in *Plasmodium falciparum.* PLoS One. 2020;15:e0236369. 10.1371/journal.pone.0236369 32702040 PMC7377462

[31] Cheng Q, Gatton ML, Barnwell J, Chiodini P, McCarthy J, Bell D, et al. *Plasmodium falciparum* parasites lacking histidine-rich protein 2 and 3: a review and recommendations for accurate reporting. Malar J. 2014;13:283. 10.1186/1475-2875-13-283 25052298 PMC4115471

[32] World Health Organization. Response plan to *pfhrp2* gene deletions. Geneva: The Organization; 2019.

[33] Lee N, Baker J, Andrews KT, Gatton ML, Bell D, Cheng Q, et al. Effect of sequence variation in *Plasmodium falciparum* histidine- rich protein 2 on binding of specific monoclonal antibodies: Implications for rapid diagnostic tests for malaria. J Clin Microbiol. 2006;44:2773–8. 10.1128/JCM.02557-05 16891491 PMC1594627

[34] Lee N, Gatton ML, Pelecanos A, Bubb M, Gonzalez I, Bell D, et al. Identification of optimal epitopes for *Plasmodium falciparum* rapid diagnostic tests that target histidine-rich proteins 2 and 3. J Clin Microbiol. 2012;50:1397–405. 10.1128/JCM.06533-11 22259210 PMC3318543

[35] Gamboa D, Ho MF, Bendezu J, Torres K, Chiodini PL, Barnwell JW, et al. A large proportion of *P. falciparum* isolates in the Amazon region of Peru lack *pfhrp2* and *pfhrp3*: implications for malaria rapid diagnostic tests. PLoS One. 2010;5:e8091. 10.1371/journal.pone.0008091 20111602 PMC2810332

[36] Golassa L, Messele A, Amambua-Ngwa A, Swedberg G. High prevalence and extended deletions in *Plasmodium falciparum hrp2/3* genomic loci in Ethiopia. PLoS One. 2020;15:e0241807. 10.1371/journal.pone.0241807 33152025 PMC7644029

[37] Alemayehu GS, Blackburn K, Lopez K, Cambel Dieng C, Lo E, Janies D, et al. Detection of high prevalence of *Plasmodium falciparum* histidine-rich protein 2/3 gene deletions in Assosa zone, Ethiopia: implication for malaria diagnosis. Malar J. 2021;20:109. 10.1186/s12936-021-03629-x 33622309 PMC8095343

[38] Girma S, Cheaveau J, Mohon AN, Marasinghe D, Legese R, Balasingam N, et al. Prevalence and epidemiological characteristics of asymptomatic malaria based on ultrasensitive diagnostics: a cross-sectional study. Clin Infect Dis. 2019;69:1003–10. 10.1093/cid/ciy1005 30475992

[39] Willie N, Mehlotra RK, Howes RE, Rakotomanga TA, Ramboarina S, Ratsimbasoa AC, et al. Insights into the performance of SD Bioline Malaria Ag P.f/Pan rapid diagnostic test and *Plasmodium falciparum* histidine-rich protein 2 gene variation in Madagascar. Am J Trop Med Hyg. 2018;98:1683–91. 10.4269/ajtmh.17-0845 29557337 PMC6086193

[40] Baker J, Ho MF, Pelecanos A, Gatton M, Chen N, Abdullah S, et al. Global sequence variation in the histidine-rich proteins 2 and 3 of *Plasmodium falciparum*: implications for the performance of malaria rapid diagnostic tests. Malar J. 2010;9:129. 10.1186/1475-2875-9-129 20470441 PMC2893195

[41] Plucinski MM, Dimbu PR, Fortes F, Abdulla S, Ahmed S, Gutman J, et al. Posttreatment HRP2 clearance in patients with uncomplicated *Plasmodium falciparum* malaria. J Infect Dis. 2018;217:685–92. 10.1093/infdis/jix622 29220497 PMC11023016

[42] Koita OA, Doumbo OK, Ouattara A, Tall LK, Konaré A, Diakité M, et al. False-negative rapid diagnostic tests for malaria and deletion of the histidine-rich repeat region of the *hrp2* gene. Am J Trop Med Hyg. 2012;86:194–8. 10.4269/ajtmh.2012.10-0665 22302847 PMC3269266

