# Supplementary material for: Plasmodium falciparum pfhrp2 and pfhrp3 Gene Deletions from Persons with Symptomatic Malaria Infection in Ethiopia, Kenya, Madagascar, and Rwanda
Source: Emerg Infect Dis. 2022 Mar;28(3):608–16. doi: 10.3201/eid2803.211499 (PMC8888236; doi:10.3201/eid2803.211499)
Supplement: Appendix — Additional information about Plasmodium falciparum pfhrp2 and pfhrp3 gene deletions from persons with symptomatic malaria infection in Ethiopia, Kenya, Madagascar, and Rwanda. [file 21-1499-Techapp-s1.pdf]

Article DOI: https://doi.org/10.3201/eid2803.211499

# *Plasmodium falciparum pfhrp2* and *pfhrp3* Gene Deletions from Persons with Symptomatic Malaria Infection in Ethiopia, Kenya, Madagascar, and Rwanda

## Appendix

### Whole Blood Elution from Dried Blood Spots

To rehydrate blood samples, a 6mm filter paper punch from each dried blood spot (≈10 µL whole blood) was placed into 200 µL of elution buffer: phosphate-buffered saline (PBS) pH 7.2, 0.3% Tween-20, 0.5% casein, 0.5% bovine serum albumin, 0.5% polyvinyl alcohol, 0.8% polyvinlypyrrolidine, 0.02% sodium azide, and 3 µg/mL of *E. coli* lysate (to prevent nonspecific binding). This provided a 1:20 dilution of whole blood that was used for the immunoassay.

### Assay Reagent Preparation and Multiplex Antigen Detection Assay

Magnetic microbeads (xMAP, Luminex Corporation, https://www.luminexcorp.com) were covalently bound to antigen capture antibodies by the Luminex antibody coupling kit according to manufacturer’s instructions. For 1 milliliter of microbeads ($12.5\times10^6$ beads) antibody coupling concentrations were anti-pan-*Plasmodium* aldolase antibody (pAldolase, 12.5 µg; Abcam, https://www.abcam.com); anti-pan-*Plasmodium* lactate dehydrogenase antibody (pLDH, 12.5 µg of clone M1209063; Fitzgerald, https://www.fitzgerald-fii.com); anti-P. falciparum HRP2 (20 µg, clone MPFG-55A; ICL Labs, https://www.icllab.com). Detection antibodies were also prepared in advance by biotinylating (EZ-link Micro Sulfo-NHS-Biotinylation Kit; ThermoFisher Scientific, https://www.thermofisher.com) according to manufacturer’s instructions. Final prepared dilution of detection antibodies was 1.0 mg/mL and for antimalarial antigen specific antibodies as follows: pAldolase (Abcam), pLDH (M86550,

Fitzgerald), HRP2 (1:1 antibody mixture of MPFG-55A and MPFM-55A, ICL Labs). All reagents were stored at 4°C until use in the immunoassay.

Assay reagents were diluted in buffer containing 0.1M PBS (pH 7.2), 0.05% Tween-20, 0.5% bovine serum albumin, and 0.02% sodium azide. For all wash steps, the assay plate was affixed to a handheld magnet (Luminex Corporation) and then gently tapped for 2 minutes to allow bead magnetization before evacuation of liquid and washing with 150 μL PBS and 0.05% Tween-20. The four bead regions were combined in dilution buffer (in a reagent trough) and pipetted onto a 96-well assay plate (BioPlex Pro, BioRad, https://www.bio-rad.com) at a quantity of ≈800 beads per region. Plates were washed twice, and 50 μL of controls or samples was pipetted into appropriate wells. Following 90-minute gentle shaking at room temperature protected from light, plates were washed 3 times. A mixture of detection antibodies was prepared in dilution buffer (pAldolase at 1:2000, all others at 1:500) and 50 μL added to each well for a 45-minute incubation. After 3 washes, 50 μL of streptavidin-phycoerythrin (at 1:200, Invitrogen) was added for a 30-minute incubation. Plates were washed 3 times, and 50 μL dilution buffer was added to each well for 30-minute incubation. Plates were then washed once and beads resuspended in 100 μL PBS. After brief shaking, plates were read on a MAGPIX machine (Luminex Corporation) with a target of 50 beads per region. The median fluorescence intensity value was generated for all beads collected for each region by assay well. Subtraction of the assay signal from wells with dilution buffer blank provided the median fluorescence intensity minus background value (MFI-bg) used for analyses. Positive and negative controls were included on each assay plate to ensure valid assay results.

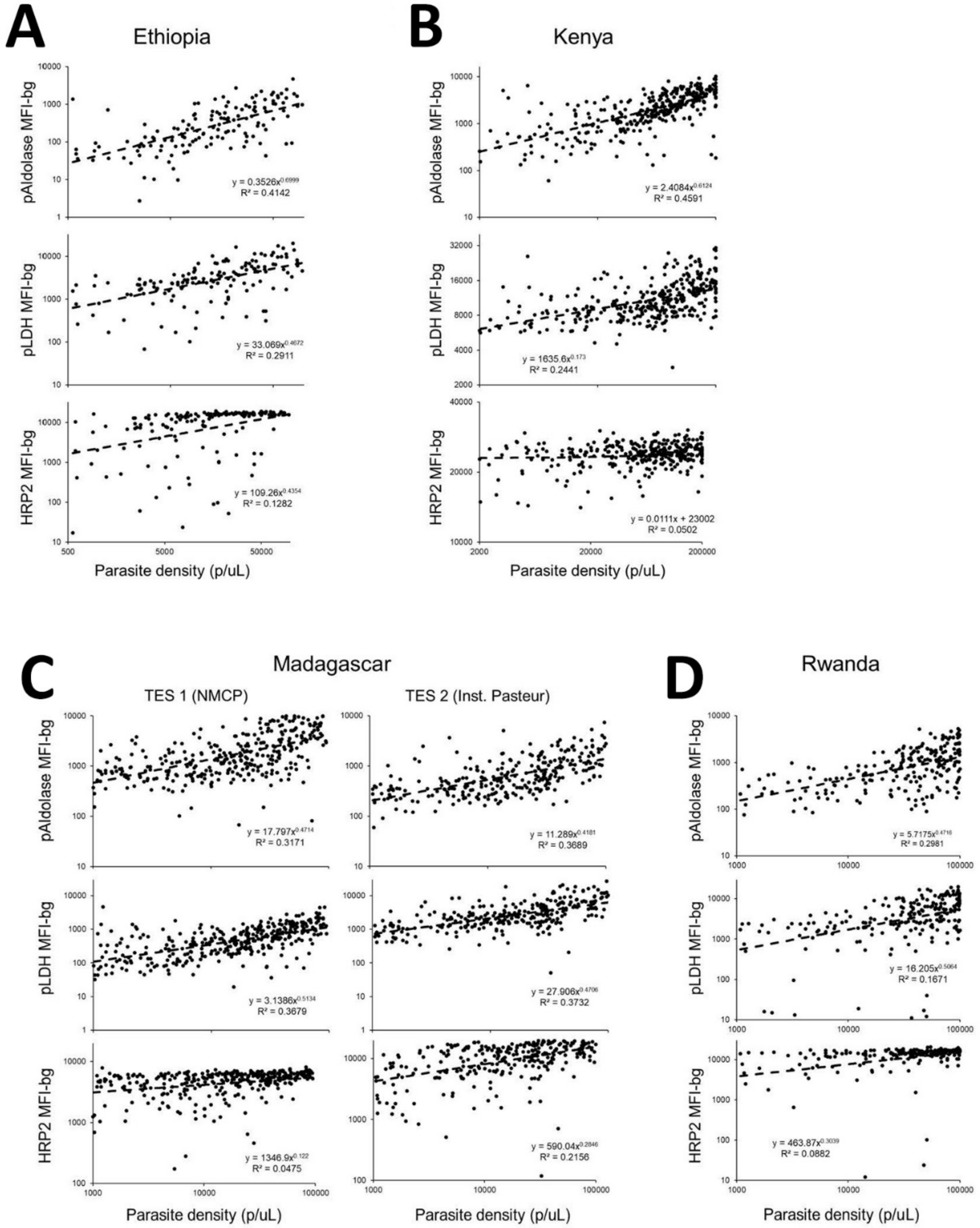


**Appendix Figure 1.** Parasite density by microscopy versus assay signal for the 3 antigens measured in *Plasmodium falciparum pfhrp2* and *pfhrp3* gene deletions from persons in therapeutic efficacy studies in Ethiopia, Kenya, Madagascar, and Rwanda. Plots shown for samples from A) Ethiopia, B) Kenya, C) Madagascar, and D) Rwanda. Each plot displays regression as a hashed line with regression estimates. HRP2, histidine-rich protein 2; MFI-bg, median fluorescence intensity minus background; pAldolase, pan-*Plasmodium* aldolase; pLDH, pan-*Plasmodium* lactate dehydrogenase.

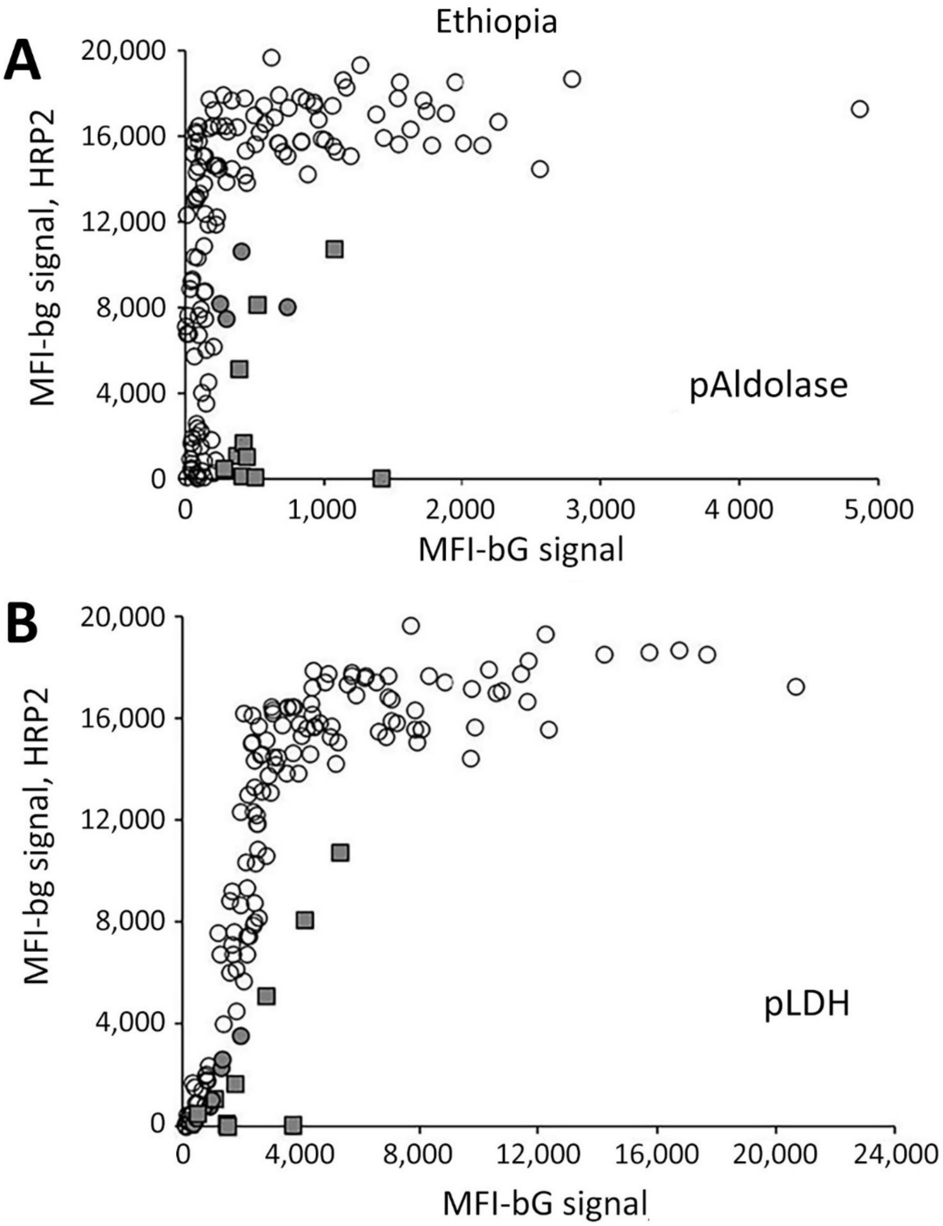


**Appendix Figure 2.** Specimens selected for genotyping from therapeutic efficacy study in Ethiopia because of atypical association of HRP2 with pAldolase or pLDH assay signal. Scatterplots compare A) pAldolase or B) pLDH assay signal with HRP2 assay signal. Shaded circles indicate samples selected for genetic assays on the basis of 1 pan-*Plasmodium* marker, and shaded squares indicate samples selected on the basis of both pan-*Plasmodium* markers.

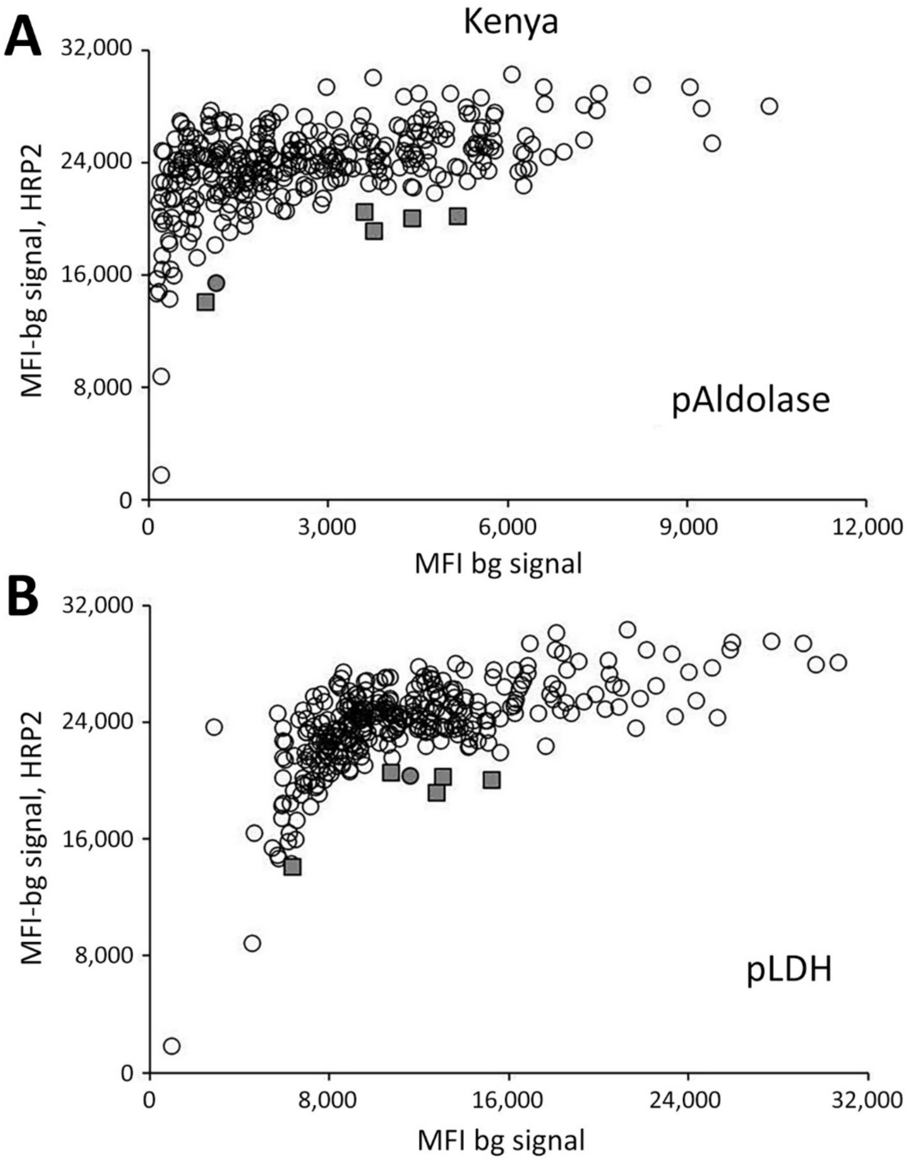


**Appendix Figure 3.** Specimens selected for genotyping from a therapeutic efficacy study in Kenya because of atypical association of HRP2 with pAldolase or pLDH assay signal. Scatterplots compare A) pAldolase or B) pLDH assay signal with HRP2 assay signal. Shaded circles indicate samples selected for genetic assays on the basis of 1 pan-*Plasmodium* marker, and shaded squares indicate samples selected on the basis of both pan-*Plasmodium* markers.

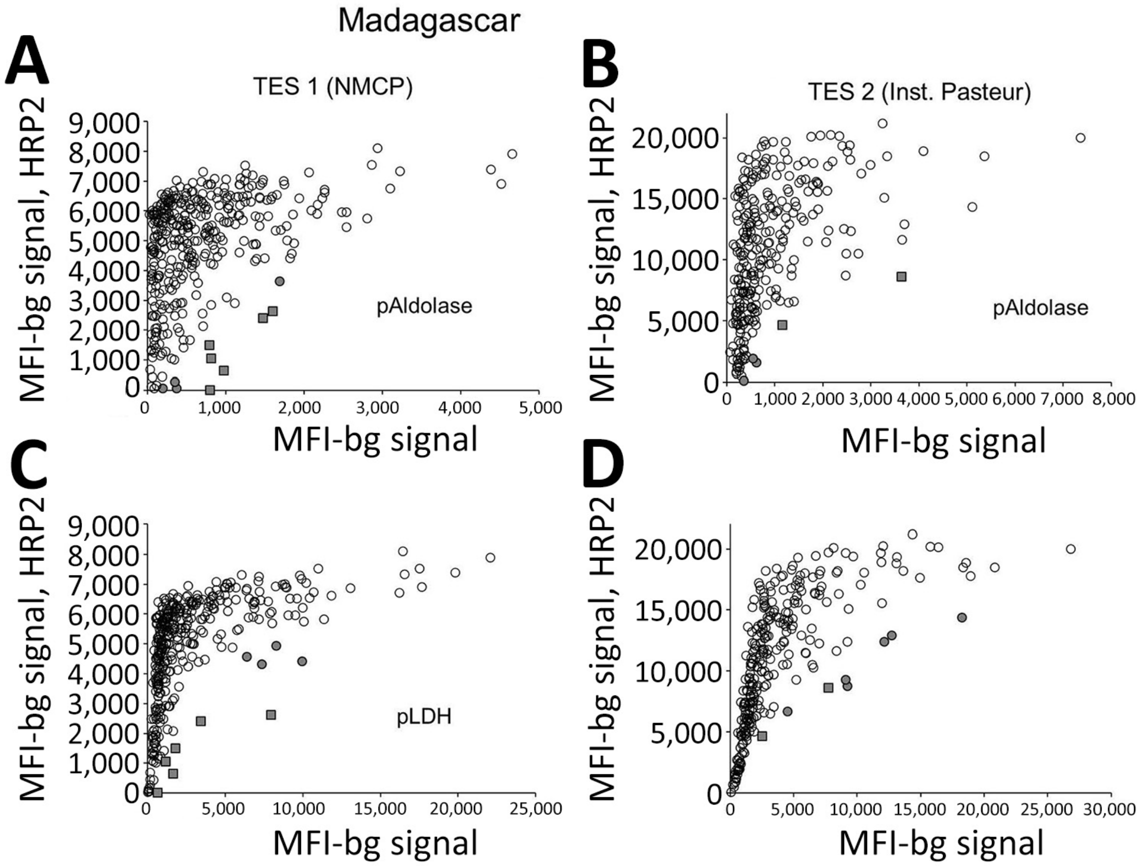


**Appendix Figure 4.** Specimens selected for genotyping from therapeutic efficacy studies (TESs) in Madagascar because of atypical association of HRP2 with pAldolase or pLDH assay signal. Scatterplots compare A) pAldolase or B) pLDH assay signal with HRP2 assay signal. Shaded circles indicate samples selected for genetic assays on the basis of 1 pan-*Plasmodium* marker, and shaded squares indicate samples selected on the basis of both pan-*Plasmodium* markers. TES 1 indicates the study initiated by the Madagascar National Malaria Control Program, and TES 2 indicates the study performed by the Institute Pasteur.

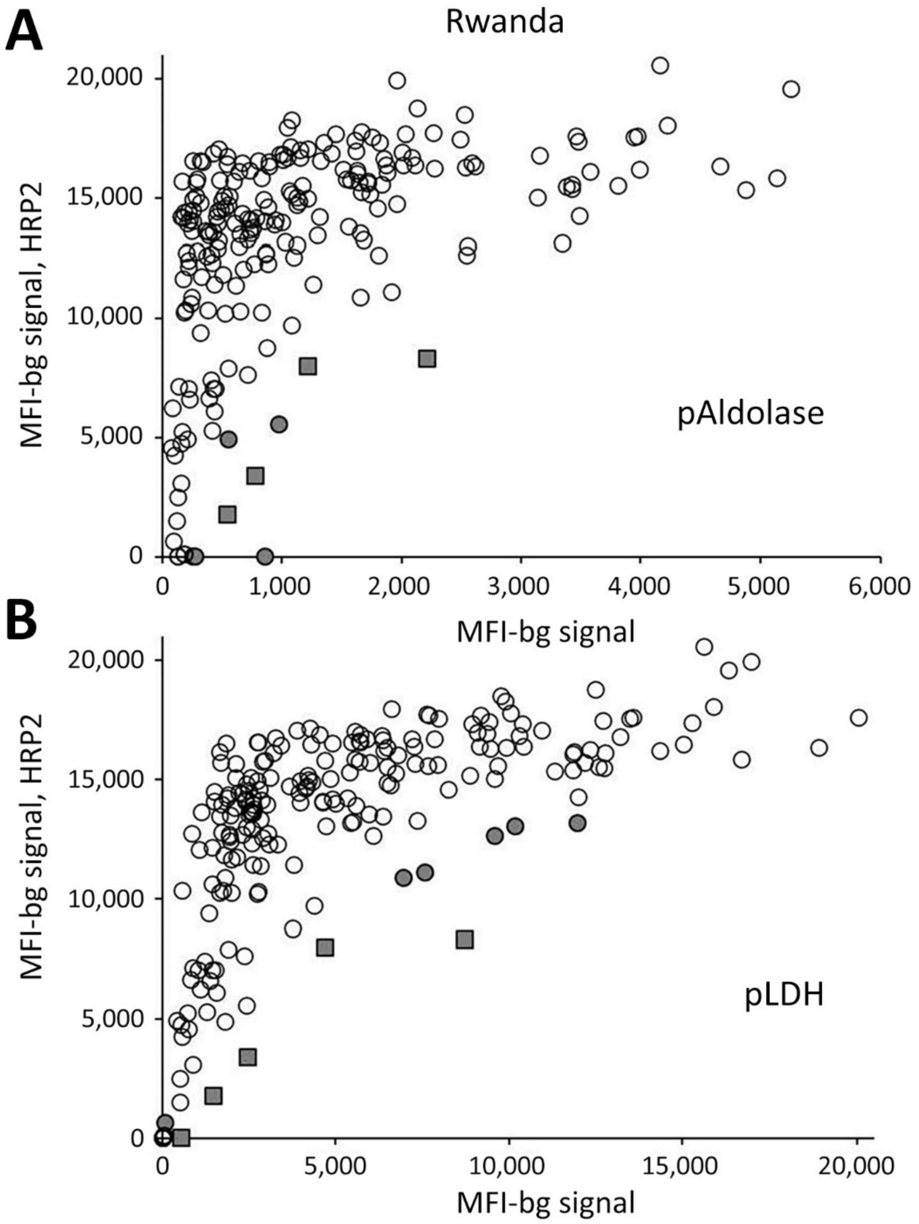


**Appendix Figure 5.** Specimens selected for genotyping from a therapeutic efficacy study in Rwanda because of atypical association of HRP2 with pAldolase or pLDH genetic assays. Scatterplots compare A) pAldolase or B) pLDH assay signal with HRP2 assay signal. Shaded circles indicate samples selected for genetic assays on the basis of 1 pan-*Plasmodium* marker, and shaded squares indicate samples selected on the basis of both pan-*Plasmodium* markers.
